# Selection of reference genes is critical for miRNA expression analysis in human cardiac tissue. A focus on atrial fibrillation

**DOI:** 10.1038/srep41127

**Published:** 2017-01-24

**Authors:** Michela Masè, Margherita Grasso, Laura Avogaro, Elvira D’Amato, Francesco Tessarolo, Angelo Graffigna, Michela Alessandra Denti, Flavia Ravelli

**Affiliations:** 1 Department of Physics, University of Trento, Trento, Italy; 2 Centre for Integrative Biology, University of Trento, Trento, Italy; 3 Healthcare Research and Innovation Program (IRCS-PAT), Bruno Kessler Foundation, Trento, Italy; 4 Department of Industrial Engineering, University of Trento, Trento, Italy; 5 Division of Cardiac Surgery, Santa Chiara Hospital, Trento, Italy

## Abstract

MicroRNAs (miRNAs) are emerging as key regulators of complex biological processes in several cardiovascular diseases, including atrial fibrillation (AF). Reverse transcription-quantitative polymerase chain reaction is a powerful technique to quantitatively assess miRNA expression profile, but reliable results depend on proper data normalization by suitable reference genes. Despite the increasing number of studies assessing miRNAs in cardiac disease, no consensus on the best reference genes has been reached. This work aims to assess reference genes stability in human cardiac tissue with a focus on AF investigation. We evaluated the stability of five reference genes (U6, SNORD48, SNORD44, miR-16, and 5S) in atrial tissue samples from eighteen cardiac-surgery patients in sinus rhythm and AF. Stability was quantified by combining BestKeeper, delta-*C*_*q*_, GeNorm, and NormFinder statistical tools. All methods assessed SNORD48 as the best and U6 as the worst reference gene. Applications of different normalization strategies significantly impacted miRNA expression profiles in the study population. Our results point out the necessity of a consensus on data normalization in AF studies to avoid the emergence of divergent biological conclusions.

MicroRNAs (miRNAs) are small (approximately 22 base pairs), single stranded, non-coding RNA molecules that finely regulate gene expression at the post-transcriptional level. miRNAs are involved in a variety of physiological and pathophysiological processes, which range from cell development and differentiation to apoptosis and oncogenesis^1^. Compelling evidence supports the role of miRNAs in normal cardiac development and in cardiac disease. Distinctive miRNAs signatures have been associated with heart failure, cardiac hypertrophy, and myocardial infarction^2^,^3^,^4^. More recently attention has been posed on the role of miRNAs as molecular determinants of atrial fibrillation (AF). AF is the most common sustained cardiac arrhythmia in the clinical practice, with 33.5 millions of people affected in 2010 and about 5 millions of new cases each year^5^. AF is associated with pronounced cardiovascular morbidity and mortality, mostly due to an increased risk of stroke. AF is a multi-factorial disease, supported by altered electrophysiological and structural factors^6^. A growing body of works suggests miRNAs to act as potential mediators in the electrophysiological and structural remodeling of the atria maintaining AF^7^,^8^,^9^.

The regulatory function of miRNAs in cardiac disease and AF supports their utilization as prognostic and predictive biomarkers as well as therapeutic targets. This requires, however, a reliable and quantitative assessment of miRNA expression. Although different methodologies can be applied to evaluate miRNA expression, reverse transcription-quantitative polymerase chain reaction (RT-qPCR) remains the gold-standard for a specific detection of selected sets of miRNAs^10^,^11^. Normalization of expression levels is a crucial step to ensure accurate and suitable quantification of PCR data^12^,^13^,^14^. Normalization is aimed to differentiate true biological variations, explaining the investigated phenotype, from non-specific experimentally-induced alterations. Indeed factors, such as sample collection and preservation, amount of raw material, enzyme efficiency, RNA integrity, can artefactually alter expression levels. To date normalization by one or a set of internal reference genes is generally accepted to normalize miRNA expression^12^. Ideal reference genes should show no (or minimal) expression variation in the tissue or cells under investigation, or in response to experimental treatment/disease condition. Since no normalization standard has been proven to be ideal yet, it is essential to verify the expression stability of putative normalizers in each experimental setup, and in relation to the specific tissue, species, and disease under investigation. Despite the growing number of studies investigating miRNA expression in human AF^7^,^8^,^9^, at present no consensus exists on the reference genes for human atrial tissue samples. This may limit study comparison and, most importantly, lead to ambiguous data interpretation and misleading biological conclusions.

This study aimed to quantitatively assess the performance of five reference genes of different RNA classes (5S ribosomal RNA (rRNA), hsa-miR-16-5p, U6 small nuclear RNA (snRNA), SNORD44 and SNORD48 small nucleolar RNAs (snoRNAs)), previously adopted for miRNA normalization in the cardiac tissue (Table 1). The stability of reference genes was assessed on human atrial tissue samples from cardiac surgery patients in normal sinus rhythm (SR) and AF. Indeed cardiac surgery constitutes the most common experimental set-up for the study of miRNA regulation in human AF. Performance was quantified by combining multiple gold-standard statistical tools (BestKeeper^15^, GeNorm^16^, NormFinder^17^ and the comparative delta*-C*_*q*_ method^18^), which assess different aspects involved in the concept of gene “stability”^13^. Finally, the impact of adopting different normalization strategies was demonstrated in the exemplifying case of the quantification of miR-499a-5p expression in the study population.

## Results

### RNA quantity and integrity

Quantity and integrity data of the total RNA among atrial tissue samples are reported in Supplementary Table S1. The extracted amount of total RNA varied among samples with a median concentration of 117.3 ng/μl (interquartile range (IQR): 91.4–246.2 ng/μl). Integrity was adequate for the analysis in all the samples, with a median value of 7.85 (IQR: 7.0–8.2).

### Reference gene stability

#### Best Keeper

**Figure 1:**
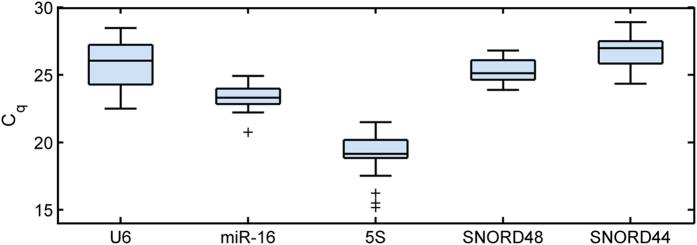
qPCR quantification cycle (*C*_*q*_) values of the five reference genes in the atrial tissue sample dataset. For each distribution values are given as median (solid line), interquartile range (IQR, box), lower and superior adjacent values at 1.5 × IQR (whiskers), and outliers (black plus sign markers).

The distributions of the qPCR quantification cycle (*C*_*q*_) values of the five reference genes over the whole sample set are shown in Fig. 1, while the descriptive statistics given by BestKeeper are reported in Table 2. Reference genes showed different expression values and variability levels. 5S showed the largest expression (mean *C*_*q*_ = 18.97), while SNORD44 was the least expressed (mean *C*_*q*_ = 26.77). In terms of variability, miR-16 and SNORD48 displayed the lowest standard deviation (SD) values of 0.70 and 0.72, respectively. Conversely, 5S and U6 showed variability levels beyond the limit of acceptance for reliable normalizers (>1). Correlation analysis was performed between each pair of reference genes and between each reference gene and the BestKeeper Index (BKI). In particular, BKI (5) was calculated including all reference genes, while BKI (3) was obtained excluding the two genes with unacceptable variability (U6 and 5S). The analysis, shown in Table 2 and in Supplementary Table S2, pointed out the existence of significant correlations for SNORD48, SNORD44 and 5S when compared to one another, and for U6 when compared to 5S, while miR-16 did not correlate with any other reference gene. Consistently, all reference genes but miR-16 correlated with BKI (5). When compared with BKI (3), SNORD48 and SNORD44 showed the highest correlations (r > 0.8 and p < 0.001), while miR-16 displayed a lower, non significant correlation value (r = 0.45, p = 0.06). Considering both variability and correlation information, SNORD48 showed the best performance, combining a low SD value with high correlation values.

#### GeNorm

**Figure 2:**
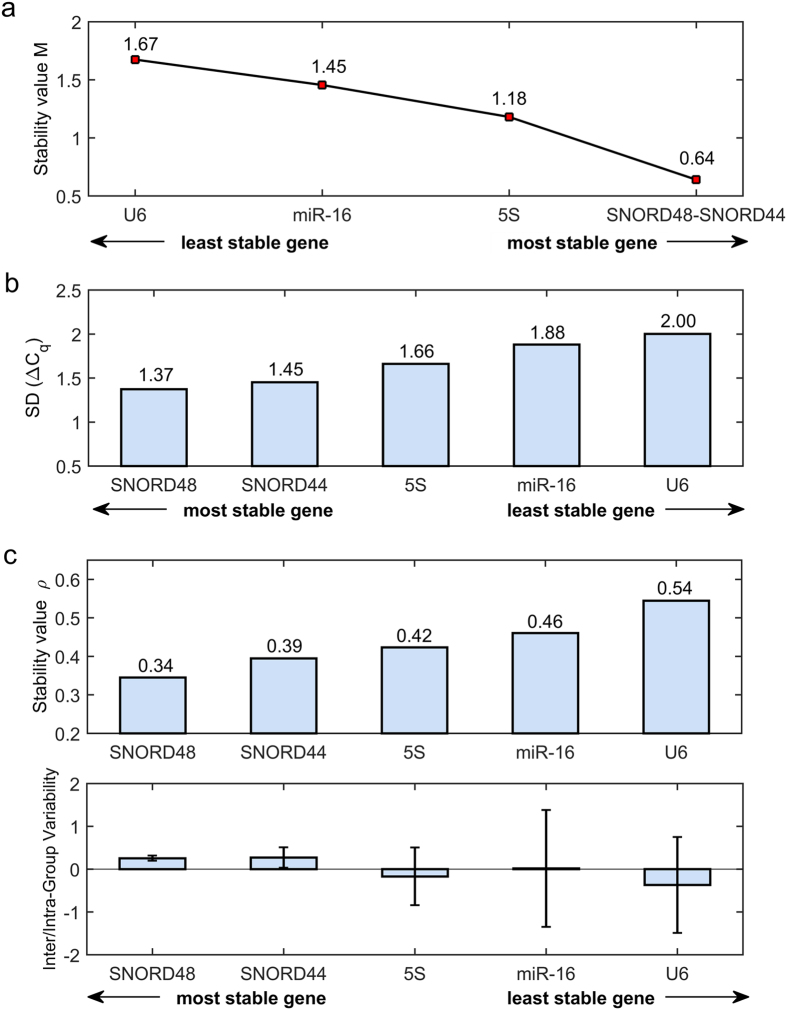
Evaluation of gene expression stability in the atrial tissue sample dataset calculated by: GeNorm (**a**), comparative delta*-C*_*q*_ method (**b**), and NormFinder (**c**). (**a**) Step-wise exclusion of the least stable reference genes, based on *M*-stability index. (**b**) Average variability (SD) of the *C*_*q*_ differences (*ΔC*_*q*_) calculated for each reference gene versus the remaining genes. (**c**) In the upper panel, overall stability index (ρ) of each candidate gene and, in the lower panel, corresponding intergroup (box) and intra-group (whiskers) variability. Note that genes are ranked right to left from the most to the least stable in panel a, and left to right in panels (**b** and **c**). See text for details.

The analysis of gene stability performed by GeNorm is reported in Fig. 2a. The procedure of step-wise exclusion of the candidate genes indicated U6 as the worst reference gene (*M*-value = 1.67), and identified SNORD48 and SNORD44 as the most stable pair of genes (*M*-value = 0.64). Pairwise variation analysis showed no combination of N > 2 candidate genes leading to a decrease in variation <0.15. The GeNorm manual suggests the use of minimum the three most stable reference genes in such situations.

#### Comparative delta-C_q_ method

Similarly to GeNorm, the comparative delta-*C*_*q*_ method (Fig. 2b) indicated U6 and miR-16 as the least stable genes, since they displayed the highest average SD of *C*_*q*_ differences for pairwise comparisons (2.00 and 1.88, respectively). The most stable genes were SNORD48 and SNORD44 with average SD of 1.37 and 1.45, respectively.

#### NormFinder

The stability analysis performed by NormFinder is displayed in Fig. 2c in terms of stability index ρ (upper panel) and inter-group (box) and intra-group (whiskers) variations (lower panel). The worst overall performance was shown by U6 (ρ = 0.54), which displayed large values of both inter-group (−0.37) and intra-group variability (1.12). Overall performance was low also for 5S and miR-16 (ρ = 0.42 and 0.46), mainly due to high intra-group variability values (0.67 and 1.36). Best overall performance was observed for SNORD48 (ρ = 0.34), which displayed intermediate inter-group variation (0.25) and the smallest intra-group variability (0.06).

The average stability index *ρ*_*A*_, calculated for each pair of reference genes, showed that stability could be improved by using combination of reference genes with intergroup variability of opposite sign. In particular, combination of either of SNORD48 and SNORD44 with U6 led to an average stability index of *ρ*_*A*_ = 0.27, while combination with 5S led to a stability index of *ρ*_*A*_ = 0.29.

**Figure 3:**
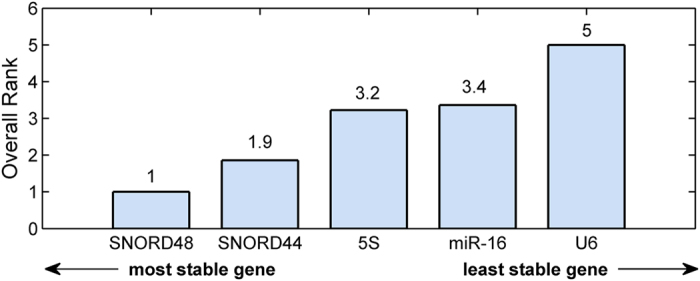
Overall ranking of the five reference genes. The overall positioning is calculated as the geometric mean of the rankings obtained by BestKeeper, GeNorm, comparative delta*-C*_*q*_, and NormFinder statistical tools. Genes are ordered left to right from the most to the least stable. SNORD48 displayed the best overall ranking.

The comprehensive ranking of gene stability obtained by combining the four analyses (Fig. 3 and Table 3) assessed SNORD48 as the most stable gene (best performance according to all analyses), followed by SNORD44, 5S, and miR-16. U6 was the least stable gene, displaying the worst performance according to all analyses.

### Impact of normalization strategy on miR-499a-5p profiling

**Figure 4:**
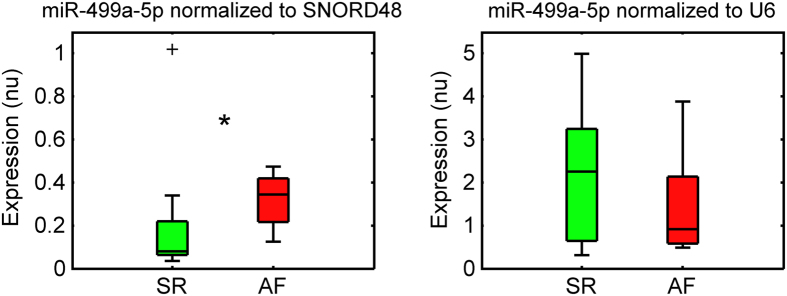
Effects of normalization strategy on miRNA expression profile. Normalized expressions of miR-499a-5p in sinus rhythm (SR, green box) versus atrial fibrillation (AF, red box) patients, calculated using the best (SNORD48, left) and the worst (U6, right) reference gene. For each distribution values are given as median (solid line), interquartile range (IQR, box), lower and superior adjacent values at 1.5 × IQR (whiskers), and outliers (black plus sign markers). nu, normalized units. *p < 0.05.

The effects of normalization strategy on target miRNA profiles were evaluated in the exemplifying case of miR-499a-5p. The expression levels in AF versus SR groups were computed using either the best (SNORD48) and worst (U6) normalizer. Results in Fig. 4 show that miR-499a-5p expression levels in the two groups were strongly affected by the normalization process. When normalizing to SNORD48, a significant (p < 0.05) overexpression of miR-499a-5p was observed in the AF versus SR group (left panel). Conversely, normalization by U6 led to an increased variability of expression and the loss of any subgroup expression difference (right panel).

## Discussion

This study assessed for the first time the stability properties of putative normalizers for miRNA expression profiling in human atrial tissue, with focus on the study of AF. By a multi-technique quantitative approach we demonstrated the poor performance of widely-used normalizers, such as U6, in this specific experimental context, and suggested a potential suitable alternative in the small nucleolar RNA SNORD48. The application of different normalization strategies in the exemplary case of miR-499a-5p assessment pointed out the criticality of the choice of reference genes to obtain reliable information on miRNA deregulation in AF.

The advent of RT- qPCR and qPCR has opened the possibility of a quantitative assessment of miRNA expression profiles. The higher sensitivity of the technique has fixed higher standard for reference gene variability, which should have prompted a re-evaluation of previously used normalization strategies. A large number of studies have been carried out concerning the validation of reference genes in different tissue and cell types^19^,^20^,^21^,^22^,^23^,^24^. Nonetheless, studies on the best normalization strategy in cardiac tissue remain sparse^25^,^26^,^27^, and, to the best of our knowledge, no previous study has been performed to identify appropriate reference genes in the human atrial tissue for the study of AF. The lack of a general consensus on the best normalization strategy in this context has significant impact, considering the large increase in the number of studies analyzing the relationship between miRNA and AF in the last five years^7^,^8^,^9^. A variety of normalization strategies has been applied in different studies, which however were not validated in the specific experimental context of application^28^,^29^. The absence of a common normalization approach and the use of unvalidated reference genes may not only hinder the comparison of results in different studies, but also call into question the reliability and biological meaning of these results.

The present study addressed the issue of miRNA normalization in the atrial tissue of SR and AF patients by an empirical and quantitative approach. We evaluated the performance of a set of five reference genes of different RNA classes (small nuclear/nucleolar RNAs, ribosomal RNA and miRNA), which have been widely used in different contexts, including AF. Since we focused on miRNA normalization, messenger RNA (mRNA) reference genes, such as GAPDH, were excluded from our set of reference genes. Indeed, being much bigger in size than miRNAs, mRNAs might differ from miRNAs in terms of stability, efficiency of extraction, reverse transcription and PCR amplification. The quantitative evaluation of reference genes was based on the combination of four complementary statistical approaches, which are currently considered the gold standard for the selection of appropriate reference genes for normalization in gene expression experiments involving qPCR^13^. The multi-technique evaluation of reference genes allowed us to take into account different aspects related to the concept of gene stability, such as overall variability, similarity and correlation of expression patterns, inter/intra-group variability. Previous studies showed that statistical approaches, based on different definitions of stability, may sometimes lead to different ranking of reference genes^30^. Thus an overall evaluation of the performance by combined measures is desirable^31^. On the other hand, the fact that in our analysis four independent algorithms agreed in ranking the most and least stable reference genes adds a level of consistency to the obtained results.

The multi-technique approach provided the following ranking of the candidate reference genes, from the most to the least stable: SNORD48, SNORD44, 5S, miR-16, U6. Small nuclear/nucleolar RNAs, such as U6, SNORD44 and SNORD48, have been commonly used for miRNA normalization, thanks to their RNA stability and abundant expression. Nonetheless, in contrast with its frequent use as normalizer in different contexts, including the study of AF^28^,^32^, our validation methods consistently ranked U6 as the worst reference gene of the set. Indeed it displayed the highest variability across the entire population compared to the other transcripts, the highest *M*-value and intergroup variability, suggesting its unsuitability as reference gene in human AF. Instability of U6 has been previously reported in samples from hepatic and liver tissues^20^, renal cell carcinomas^23^, endometrial^22^ and prostate^24^ cancer tissues. In addition, a disease-specific dysregulation of U6 has been suggested by Benz *et al*.^33^. They observed a high variability of U6 expression in the serum of healthy volunteers, intensive care unit and liver fibrosis patients, and a significant correlation of U6 with established inflammation markers^33^. Since an inflammation state may be linked to cardiac valve disease leading to cardiac surgery^34^, U6 deregulation with inflammation might partially explain the bad performance of the reference gene in atrial samples from a cardiac-surgery setting. In contrast with these negative results, U6 was assessed among the most stable control genes in the rat heart^26^. Differences in stability may be related to differences in species and experimental setting and/or to pathology-specific effects. This points out the inappropriateness of simply transposing reference genes from study to study and the necessity of an empirical case-by-case validation.

Differently from U6, the two nucleolar RNAs, SNORD44 and SNORD48, displayed the best performance of all candidate genes, ranking second and first, respectively. In particular, SNORD48 showed expression levels higher than SNORD44, low overall variability of expression and intra-group variation, and high correlation and similarity of expression with the other reference genes. These features resulted in the best scores according to all algorithms. Consistently with our results, SNORD48, SNORD75 and SNORD44 were scored among the most stably and equivalently expressed reference genes in tissue samples of endometroid endometrial carcinoma patients and normal samples, displaying better performance than miR-16 and U6^22^. In addition, SNORD48 resulted the most stably expressed reference gene in a large set of forensically relevant organ tissues^19^, as well as in renal cell carcinoma^23^.

Our study revealed intermediate performance of the ribosomal RNA 5S and of miR-16. Consistently with its structural role in the large ribosomal subunit, 5S displayed the highest expression levels of all candidate genes. Expression levels significantly larger than target RNAs may raise concerns on the use of 5S as a normalizer, since it may be complex to quantify the ribosomal RNA and a rare target in the same RNA dilution. In addition we observed a large variability in the overall expression of 5S, which may further reduce its performance as normalizer. Conversely, the use of miRNAs, such as miR-16, as reference genes in miRNA normalization may be supported, since being of the same RNA class, they should have similar efficiency in extraction, reverse transcription and PCR amplification. In our analysis miR-16 presented low overall variability and inter-group variability, but its overall performance was degraded by a high intra-group variation and expression patterns dissimilar from the other reference genes. The low correlation and dissimilarity in expression may be partially explained by its different RNA class and potentially different efficiency properties. Low performance of miR-16 as normalizer was reported in prostate carcinomas^24^. In addition miR-16 appeared deregulated by different cancer types^22^,^24^,^35^ and in myelodysplastic syndrome^36^.

The marked differences in performance of the tested normalization strategies had a significant impact on miRNAs profiling in AF, hindering biological implications. In the representative example of miR-499a-5p, we showed that normalization by the best reference gene pointed out differences in the expression levels between AF and SR patients, which were lost by applying the wrong normalization strategy. The observed difference in expression is in agreement with previous results in human atrial tissues from cardiac surgery patients, where miR-499a-5p resulted significantly upregulated in AF^37^. In particular, the study showed that miR-499a-5p overexpression led to a downregulation of the protein expression of the small-conductance calcium-activated potassium channel 3, which may potentially contribute to AF electrical remodeling^37^. Interestingly, in ref. 37 miRNA expression was normalized to GAPDH messenger RNA from the same preparation. The observation of similar expression profiles in presence of different normalization strategies may add further consistency to our results.

### Limitations

*C*_*q*_ values were estimated in each patient from a single tissue sample. Thus the observed variability may be related not only to the population, but also to the tissue sampling step (see Supplementary Analysis of Variability and Fig. 1S). When allowed by the clinical setting, evaluation of miRNA expression from human cardiac tissue should be performed from multiple samples in each individual to reduce overall variability^38^. As well, normalization performance may be improved by using SNORD48 in combination with other reference genes^16^, but optimal reference gene combination needs further investigation.

## Conclusion

This study pointed out the unreliable performance of reference gene U6 in AF studies involving human atrial tissue samples from cardiac surgery settings, and suggests instead the use of SNORD48 as single normalizer of miRNA expression. Our results stress the importance of testing and validating reference genes in each specific experimental and disease condition. In order to avoid the continuous emergence of divergent and contradictory conclusions in the study of miRNAs and AF, a consensus on data normalization is urged before miRNAs are further quantified using relative qPCR in human AF.

## Materials and Methods

### Sample collection

Tissue samples from the right atrial appendage were excised during open cardiac surgery in 18 patients, undergoing aortic or mitral valve replacement with extracorporeal circulation at the Santa Chiara Hospital of Trento. The patients were all males, with a mean age of 70.7 ± 10.6 years (range 44–85 years). The patients were divided into two groups: patients in normal SR (n = 11, without history of AF) and patients with AF (n = 7, documented arrhythmia >6 months before surgery). Demographic and clinical details about the population are reported in Table 4. The investigation was approved by the Ethical Committee for Clinical Experimentation of the Provincial Agency for Health Services of the Autonomous Province of Trento, and conducted according to the tenets of the Declaration of Helsinki. All patients gave written informed consent.

### Sample processing and RNA isolation

Small right atrial appendage biopsies (~30–50 mg) were flash frozen in pre-chilled liquid isopentane and stored at −80 °C until RNA isolation. Each frozen tissue sample was placed in a sterile 15 ml polypropylene tube containing 1,5 ml of pre-cooled Qiazol reagent (Qiagen, Milan, Italy) and subsequently homogenized in ice by a Polytron (Omni-TH International, Kennesaw, USA) at half speed. Total RNA was extracted using miRNeasy mini kit (Qiagen, Milan, Italy) according to the manufacturer’s protocol. RNA concentration and purity were assessed spectrophotometrically by Nanodrop ND-1000 (Thermo Scientific, Wilmington, DE, USA). RNA integrity was evaluated for each sample using Agilent 2100 Bioanalyzer (Agilent Technologies, Santa Clara, CA, USA) with RNA 6000 Nano Kit. We set the RNA Integtity Number (RIN) threshold for good RNA >5, considering the higher stability of miRNAs^39^,^40^. RNA aliquots were stored at −80 °C until use.

### Selection of candidate reference genes

Candidate reference genes were selected based on a literature survey encompassing studies on miRNA normalization in the cardiac tissue and in the study of AF. Five reference genes of different RNA classes were identified: 5S rRNA, U6 snRNA, hsa-miR-16-5p, SNORD48 and SNORD44 snoRNAs. Specifications on the five reference genes are reported in Table 1.

### Reverse transcription

The total RNA isolated from each tissue sample was reverse-transcribed using miRCURY LNA^TM^ Universal cDNA Synthesis kit II (Exiqon, Vedbaek, Denmark), containing 2 μl of Reaction Buffer 5X, 1 μl of Enzyme mix, 10 ng of RNA in a reaction volume of 10 μl, according to the manufacturer’s protocol (Exiqon: Cat. No. 203301, Version 6.1, 04/2015). The reaction conditions were: incubation at 42 °C for 60 minutes, heat-inactivation of the reverse transcriptase at 95 °C for 5 minutes, cooling and storage at 4 °C. The retrotranscription was realized by adding a poly-A tail to the mature miRNA template and synthesizing the cDNA by a poly-T primer with a 3′ degenerate anchor and a 5′ universal tag.

### qPCR

qPCR assays based on SYBR Green I were performed on the five reference genes and on hsa-miR-499a-5p according to the manufacturer’s protocol (Exiqon: Cat. No. 203403, Version 6.1, 04/2015). miRCURY LNA^TM^ PCR primers set for 5S rRNA (Assay ID: 203906; batch number: 176192), U6 snRNA (Assay ID: 203907; batch number: 164846), hsa-miR-16-5p (Assay ID: 205702; batch number: 159156), SNORD48 snoRNA (Assay ID: 203903; batch number: 171942), SNORD44 snoRNA (Assay ID: 203902; batch number: 171664), and hsa-miR-499a-5p (Assay ID: 205935; batch number: 190086) were purchased from Exiqon (Vedbaek, Denmark). hsa-miR-499a-5p was analyzed to evaluate the effects of different normalization strategies on miRNA profiling. Both forward and reverse primers were miRNA-specific and optimized with LNA^TM^. qPCR reactions were performed using ExiLENT SYBR^®^ Green master mix (Exiqon, Vedbaek, Denmark) in a CFX384 Real-Time PCR Detection System (Bio-Rad Laboratories, Milan, Italy). The 10 μl PCR reaction contained 4 μl of the diluted cDNA template, 5 μl of SYBR^®^ Green master mix and 1 μl of PCR primer mix. The reaction protocol was as follows: 95 °C for 10 minutes, followed by 40 amplification cycles at 95 °C for 10 seconds and 60 °C for 1 minute. Each sample was assessed in technical triplicates. Replicates at the qPCR step were used to ensure against a failed reaction so that the data point was not missed. qPCR no template controls (NTCs) were run to set the background level. No sign of contamination was observed. The *C*_*q*_ was determined using Bio-Rad CFX Manager (Bio-Rad Laboratories Inc., Hercules, California, US) and single threshold method. *C*_*q*_ values were estimated for each patient and gene as the average of the technical triplicates *C*_*q*_, after removal of the outliers (*C*_*q*_ values that differed more than 1 from the triplicate median). *C*_*q*_ values or 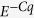 expression values (where the amplification efficiency, *E*, was assumed equal to 2) were used as inputs for subsequent stability analysis. Normalized expression for the gene of interest (GOI, miR-499a-5p) with respect to a specific reference gene (RG) was given by *E*^*−dCq*^, where *dC*_*q*_ = *C*_*qGOI*_ *−* *C*_*qRG*_.

### Assessment of reference gene stability

Gene expression stability was evaluated according to four gold-standard statistical approaches: BestKeeper^15^, GeNorm^16^, the comparative delta-*C*_*q*_ method^18^, and NormFinder^17^.

BestKeeper^15^ assumes that reliable reference genes should display low variability and similar expression patterns. Variability is assessed by computing the SD of the reference gene *C*_*q*_, and genes with SD >1 are considered inadequate. Pattern similarity is evaluated by calculating Pearson’s linear correlation coefficients (*r*) of all possible pairs of reference genes, and of the reference genes with the BestKeeper Index (BKI). This is obtained as the geometric mean of the *C*_*q*_ values of the reference genes in each sample. Genes with low SD and high correlation coefficients are the most stable.

GeNorm^16^ is based on the assumption that the expression ratio of two reference genes should be constant across samples. Gene stability is assessed computing the *M*-value, which is defined as the average pairwise variation of a particular reference gene with all other reference genes. Genes with the lowest *M*-value are the most stable. A repeated process of stepwise exclusion of the worst scoring reference gene is performed till the best pair of reference genes is identified. In addition, GeNorm computes the pairwise variation coefficient (*V*-value) to determine the optimal number of reference genes for a more accurate normalization. If the *V*-value is less or equal to a 0.15 cut-off value, it is not necessary to add further genes for normalization.

The delta-*C*_*q*_ method^18^ is based on assumptions similar to GeNorm, but pairs of reference genes are quantitatively compared in terms of the variability of their *C*_*q*_ differences (*ΔC*_*q*_) over the samples. The stability of each gene is quantified as the average SD over the pairwise comparisons with all other reference genes. Genes with the lowest average SD are the most stable.

NormFinder^17^ is based on a solid mathematical model of gene expression and statistical framework, which allows to estimate not only the overall expression variation of the candidate genes, but also the variations between sample subgroups of the same set (e.g., disease vs control subjects). For each reference gene a stability value *ρ* is computed, which takes into account both intra- and inter-group variations. Genes with the lowest *ρ-*value are the most stable. In addition, NormFinder can compute an average stability value (*ρ*_*A*_) for combinations of reference genes to assess if the average of these reference genes can improve stability with respect to the use of a single reference gene. In this study, the *ρ*_*A*_ index was computed for each combination of two reference genes.

The overall performance of the reference genes was evaluated by combining the results of the four analyses^31^. Specifically, a global ranking of the genes was obtained as the geometric mean of the rankings given by each analysis.

### Statistical analysis

Categorical variables were expressed as numbers or percentages. Statistical differences between categorical data were evaluated by Pearson’s Chi Square test. Continuous variables were given by mean ± SD or median (IQR), depending on data normality (assessed by Lilliefors test). Consistently, statistical differences between continuous variables were evaluated by unpaired Student t-test or Wilcoxon-Mann-Whitney test. A p < 0.05 was considered statistically-significant.

## Additional Information

**How to cite this article**: Masè, M. *et al*. Selection of reference genes is critical for miRNA expression analysis in human cardiac tissue. A focus on atrial fibrillation. *Sci. Rep.* **7**, 41127; doi: 10.1038/srep41127 (2017).

**Publisher's note:** Springer Nature remains neutral with regard to jurisdictional claims in published maps and institutional affiliations.

## Supplementary Material

Supplementary Material

## Figures and Tables

**Table 1:** Specifications of the five reference genes under evaluation.

Reference Gene/Alias	HUGO gene abbreviation	RNA class	Chromosome Location	NCBI reference	References
5S; r5S	RN5S1@	Ribosomal RNA (rRNA)	1q42.11-q42.13	V00589.1	Zhang Y *et al*.^41^
hsa-miR-16-5p; miR-16	MIR16-1	microRNA (miRNA)	13q14.2	LM378756.1	Nishi H *et al*.^29^ Roy S *et al*.^42^
SNORD44 U44 RNU44	SNORD44	Small nucleolar RNA (snoRNA)	1q25.1	NR_002750.2	Ferreira LR *et al*.^43^
SNORD48; U48; RNU48	SNORD48	Small nucleolar RNA (snoRNA)	6p21.33	NR_002745	Sauer E *et al*.^19^
U6; RNU6	Not available from the provider	Small nuclear RNA (snRNA)	Not available from the provider	Not available from the provider	Satoh M *et al*.^44^ Cooley N *et al*.^28^Adam O *et al*.^45^ Villar AV *et al*.^46^García R *et al*.^47^ Song CL *et al*.^48^Liu H *et al*.^32^ Dong S *et al*.^49^

**Table 2:** BestKeeper descriptive statistics and correlation analysis of the five reference genes.

Reference Genes	U6	miR-16	5S	SNORD48	SNORD44	BKI (N = 5)	BKI (N = 3)
n. samples	18	18	18	18	18	18	18
geo Mean [C_q_]	25.72	23.30	18.88	25.29	26.73	23.81	25.07
ar Mean [C_q_]	25.79	23.32	18.97	25.30	26.77	23.83	25.08
Min [C_q_]	22.49	20.75	15.17	23.88	24.34	21.82	23.86
Max [C_q_]	28.46	24.91	21.49	26.78	28.90	25.22	26.78
SD [ ± C_q_]	1.62	0.70	1.28	0.72	1.06	0.72	0.61
CV [% C_q_]	6.29	3.01	6.76	2.84	3.97	3.02	2.44
*r* with BKI (N = 5) (p-value)	0.69 (<0.002)	0.08 (0.77)	0.92 (<0.001)	0.69 (<0.002)	0.76 (<0.001)	—	—
*r* with BKI (N = 3) (p-value)	NA	0.45 (0.06)	NA	0.85 (<0.001)	0.86 (<0.001)	—	—

Ar = arithmetic; BKI = BestKeeper Index, calculated over all reference genes (N = 5) or excluding the two genes with the highest variability (N = 3); C_q_ = qPCR quantification cycle; CV = coefficient of variation; geo = geometric; Min = minimal value; Max = maximal value; *r* = Pearson’s linear correlation coefficient; SD = standard deviation.

**Table 3:** Overall stability of candidate reference genes according to the four evaluation algorithms.

Rank	Gene	Overall Ranking	BestKeeper		GeNorm	Delta Ct	NormFinder
			SD(C^q^)	*r*, BKI (N = 3)	*M* value	SD(∆ C^q^)	ρ value
1	SNORD48	1	0.72	0.85	0.64	1.37	0.34
2	SNORD44	1.9	1.06	0.86	0.64	1.45	0.39
3	5S	3.2	1.28	NA	1.18	1.66	0.42
4	miR-16	3.4	0.70	0.45	1.45	1.88	0.46
5	U6	5	1.62	NA	1.67	2.00	0.54

Genes are ordered according to the comprehensive ranking given by the geometric mean of the rankings obtained by the four analyses. BKI = BestKeeper Index, calculated excluding the two genes with the highest variability (N = 3); C_q_ = qPCR quantification cycle; SD(ΔC_q_) = average standard deviation of C_q_ differences; NA = not assigned; *r* = Pea*r*son’s linear correlation coefficient; SD = standard deviation.

**Table 4:** Demographic and clinical description of the patient population.

	SR group (n = 11)	AF group (n = 7)	p
Age (years)	67 ± 12	76 ± 4	ns
Men, n (%)	11 (100)	7 (100)	ns
Aortic valve disease, n (%)	7 (64)	5 (71)	ns
Mitral valve disease, n (%)	1 (9)	3 (43)	ns
Coronary artery disease, n (%)	3 (27)	1 (14)	ns
Angina pectoris, n (%)	6 (55)	2 (29)	ns
Chronic obstructive pulmonary disease, n (%)	1 (9)	1 (14)	ns
Diabetes Mellitus, n (%)	2 (18)	1 (14)	ns
Hypertension, n (%)	7 (64)	7 (100)	ns
Renal insufficiency, n (%)	1 (9)	1 (14)	ns
Metabolic disease, n (%)	3 (27)	3 (43)	ns
Dilated left atrium (diameter ≥ 41 mm), n (%)	7 (64)	4 (57)	ns
LV EF, %	64.5 [62–68]	60 [53.5–68.5]	ns
Isolated AVR, n (%)	3 (27)	2 (29)	ns
Isolated MVR, n (%)	0 (0)	2 (29)	ns
Combined AVR and CABG, n (%)	6 (55)	3 (43)	ns
Combined MVR and CABG, n (%)	2 (18)	0 (0)	ns

The two patient subgroups correspond to patients in sinus rhythm (SR) or atrial fibrillation (AF). Data are numbers (n) or percentages (%), and mean ± standard deviation or median [interquartile range], as pertinent. AVR, aortic valve replacement; CABG, coronary artery bypass grafting; LV EF, left ventricular ejection fraction; MVR, mitral valve replacement; ns, non-significant.
