# Supplementary material for: Selection of reference genes is critical for miRNA expression analysis in human cardiac tissue. A focus on atrial fibrillation
Source: Sci Rep. 2017 Jan 24;7:41127. doi: 10.1038/srep41127 (PMC5259703; doi:10.1038/srep41127)

Selection of reference genes is critical for miRNA expression analysis in human cardiac tissue. A focus on atrial fibrillation

Michela Masè, Margherita Grasso, Laura Avogaro, Elvira D'Amato, Francesco Tessarolo, Angelo Graffigna, Michela Alessandra Denti, and Flavia Ravelli

Supplementary Material

Supplementary Table S1. Quantity and integrity of RNA extracted from the atrial tissue sample dataset.

	Concentration (ng/µl)	RIN number
Sample 1	512.3	7.2
Sample 2	331.1	7
Sample 3	517.2	5.2
Sample 4	246.2	5.3
Sample 5	104.4	6.9
Sample 6	55.0	8.3
Sample 7	79.8	8.6
Sample 8	267.8	8
Sample 9	91.4	8.4
Sample 10	108.9	7.7
Sample 11	139.9	7.1
Sample 12	117.3	6.5
Sample 13	117.3	8.2
Sample 14	109.6	7.7
Sample 15	131.3	8.2
Sample 16	69.8	8
Sample 17	85.8	8.4
Sample 18	156.2	8

RIN, RNA Integrity Number.

Supplementary Table S2. Correlation analysis of reference genes provided by BestKeeper. Genes are pairwise correlated one with another and with the BestKeeper Index (BKI), calculated on all reference genes (N=5), or excluding the two genes with the highest variability (N=3). For each comparison the Pearson's linear correlation coefficient *r* is reported with the corresponding *p*-value in parenthesis.

**Versus**	**U6**	**miR-16**	**5S**	**SNORD48**	**SNORD44**
**U6**	-	-	-	-	-
**miR-16**	-0.20 (0.43)	-	-	-	-
**5S**	0.64 (<0.004)	-0.12 (0.64)	-	-	-
**SNORD48**	0.15 (0.56)	-0.03 (0.91)	0.53 (0.02)	-	
**SNORD44**	0.19 (0.45)	-0.04 (0.87)	0.65 (<0.003)	0.91 (<0.001)	
**BK (N=5)**	0.69 (<0.002)	0.08 (0.77)	0.92 (<0.001)	0.69 (<0.002)	0.76 (<0.001)
**BK (N=3)**	-	0.45 (0.06)	-	0.85 (<0.001)	0.86 (<0.001)

Supplementary Analysis of Variability

The potential sources of variability observed in the C_q values of reference genes were evaluated in three additional patients (Pt.A, Pt.B, Pt.C), applying a hierarchical experimental design similar to that proposed by Tichopad et al.[1] Specifically, three atrial tissue samples were collected from each of the three patients. Each sample was retro-transcribed, and then split into three RT-qPCR aliquots (leading to nine C_q values per patient per reference gene). C_q value distributions were analyzed by nested ANOVA statistics, which estimated the variability components related to the different processing steps. The results of the analysis are indicated in Supplementary Figure S1.

Reference

1. Tichopad,A. *et al.* Design and optimization of reverse-transcription quantitative PCR experiments. *Clin. Chem.* 55, 1816-1823 (2009).

Supplementary Figure S1. Sources of variability in C_q estimates.

Left panels. C_q values of each reference gene in three representative patients (Pt.A, Pt.B, Pt.C, rows). Different colors indicate the replicate values obtained from each of the three different atrial tissue samples. Despite differences among patients, the sampling step had a major effect on the observed C_q variability with respect to that of RT-qPCR replicates. The nested ANOVA statistics applied to the three patients dataset showed that the variability component related to the sampling step ranged between 79.2% and 99.3% for the analyzed reference genes.

Right panels. Mean C_q values (C_{qm}) obtained by averaging technical replicates in each atrial tissue sample (colors). Albeit differences in the absolute C_q values, the same trend of expression as a function of the reference genes was consistently observed in the three tissue samples from each patient.

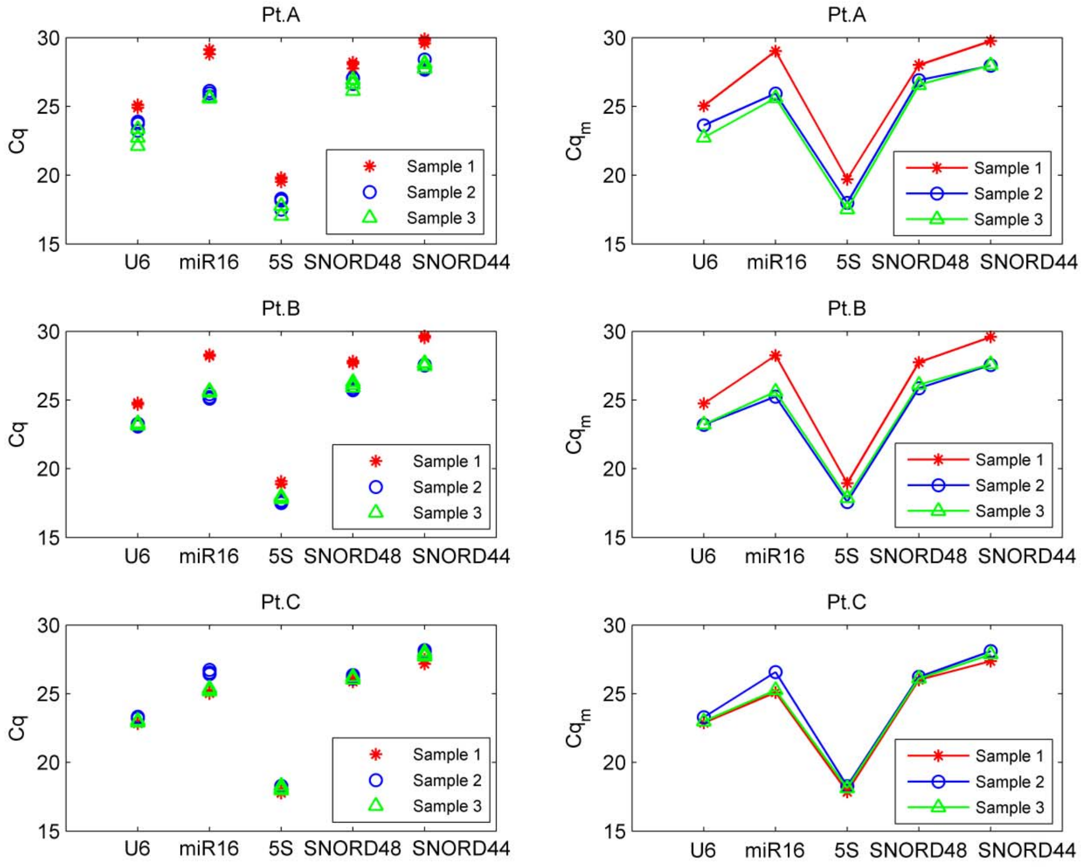

Supplement: Supplementary Material [file srep41127-s1.pdf]
